# Measuring Food Service Satisfaction amongst Residents Living in Nursing Homes—A New and Valid Person-Centered Approach

**DOI:** 10.3390/nu15030508

**Published:** 2023-01-18

**Authors:** Morgan Pankhurst, Alison Yaxley, Michelle Miller

**Affiliations:** Caring Futures Institute, College of Nursing and Health Sciences, Flinders University, Bedford Park, SA 5042, Australia

**Keywords:** food service satisfaction, nursing home, residential aged care home, psychometric properties, questionnaire, malnutrition

## Abstract

Resident satisfaction with food services contributes to health and wellbeing. Measuring resident satisfaction is important; however, the small number of existing food service satisfaction questionnaires (FSSQs) are outdated, lack rigorous psychometric testing and do not reflect the shift to person-centered care. This study aimed to develop a valid and reliable FSSQ. Content validity was established by conducting interviews with residents, a systematic literature review and consultation with an expert panel. Data from 387 residents were used to establish construct validity (Principal Components Analysis), internal consistency (Cronbach’s alpha) and temporal stability (Gwet’s AC). The result was a three factor, 25-item scale with good/excellent internal consistency: Factor One (13 items–good food/service, α = 0.896), Factor Two (seven items–resident choice/food availability α = 0.648) and Factor Three (five items–resident participation/staff assistance, α = 0.729). Temporal stability was good/very good (Gwet’s AC 0.6242–0.9799 (*p* < 0.001). This is the first FSSQ available to nursing homes that meets the COSMIN^®^ standards for excellence and incorporates person-centered care. The questionnaire is simple to use and interpret, providing food service managers with an accurate and reliable measure of resident satisfaction and assisting them in providing a meal and dining experience that supports the health and wellbeing of residents.

## 1. Introduction

Although many countries encourage and support ‘aging in place’ [1], the illness and disability common with ageing [2] mean many older adults require the specialized support of long-term residential aged care. Globally, these services have different labels including Skilled Nursing Facilities (USA), Long Term Care Homes (Canada, USA), Care Homes (UK) and Residential Aged Care Homes (Australia); however, in most countries they are often referred to as ‘nursing homes’. These facilities have traditionally operated on a medical model and provide both clinical and hospitality services for residents in their care. Clinical services are directly relatable to the health and personal care of residents such as medication, pain management, falls and pressure sores [3]. Hospitality services include the other aspects underpinning resident care such as laundry services, activities and meals [3].

The rate of malnutrition in nursing homes is persistently high, with studies suggesting approximately 50% of residents are malnourished [4,5]. Consequently, food services in a nursing home are often considered through a clinical lens with mealtimes as a vehicle for ensuring residents receive ‘adequate nutrition and hydration’ with the goal of preventing unintentional weight loss [6]. From the resident perspective, however, mealtimes become a central part of life [7,8,9,10,11,12] and, for many, are the highlight of the day [8,13,14]. Residents who experience a disappointing or unsatisfying dining experience are confronted with this reality on a daily basis [9], thereby impacting their wellbeing and quality of life [11,12,15]. A recent study suggests that when residents are dissatisfied with the food service this increases their risk of malnutrition twenty-fold [16]. It is well established that malnutrition is multifactorial [4,5,17,18]; however, logic suggests that food has no nutritional value if left uneaten and residents are far more likely to eat if presented with food they enjoy in an environment conducive to eating [19]. Therefore, understanding the food service factors that contribute to increased satisfaction and, consequently, increased consumption is necessary to address the problem.

Food service satisfaction questionnaires (FSSQs) are an effective and economical method for measuring resident satisfaction with the meals and dining; however, to be useful, they must be valid and reliable. A recent systematic literature review [20] identified three discrete consumer (resident) FSSQs intended for use in nursing homes: two from America [15,21] and one from Australia [22]. One of the aims of the review was to critically appraise the psychometric properties of these questionnaires to independently evaluate their validity and reliability. The COSMIN^®^ tool is the most comprehensive method of evaluating the results of questionnaire validation studies [23] and is recommended by the National Health and Medical Research Council as the most appropriate tool for measurement properties [24]. Critical appraisal of the existing FSSQs using the COSMIN^®^ tool showed that none have adequately established validity and reliability [20]. Additionally, the most recent FSSQ is over ten years old, during which time the aged care standards within many countries have changed to embrace a person-centered model of care [3,25,26].

In an attempt to improve resident quality of life, many countries have adopted a person-centered model of care which recognizes that food is not only necessary for optimum physical health but also plays a role in residents’ mood, comfort and wellbeing [3,25,26,27,28]. Four key concepts have been identified as important components of person-centered mealtimes: (1) providing choice and honoring preferences, (2) supporting independence, (3) showing respect and (4) promoting social interactions [19]. This research suggests that resident independence is primarily related to supporting residents to feed themselves [19]; however, it also relates to aspects of autonomy, such as being able to choose when meals are served [29] and being able to participate in everyday activities such as menu planning, peeling vegetables or folding napkins [30,31]. Indeed, these elements of person-centered care have been integrated into the Australian Aged Care Quality and Safety Standards [3]. This change is consistent with standards in other countries such as the United States of America where organizations such as the American Dietetic Association have endorsed the New Dining Practice Standards [28]. As mentioned, existing FSSQs were created when the medical model was dominant; consequently, the areas they do not adequately address are supporting resident independence, autonomy and participation. Therefore, the aim of this study was to develop, test and refine a resident FSSQ that meets the COSMIN^®^ standards for excellence and includes aspects of person-centered care.

## 2. Materials and Methods

Design and Development: The COSMIN^®^ [23] guidelines were originally developed as a quality appraisal tool; however, the benchmarks for excellence described therein can be used during questionnaire design and development. Accordingly, the guidelines were used in conjunction with a consultant statistician to determine factors such as sample size, statistical methods and reporting, thereby ensuring the final instrument would be valid and reliable.

Content validity is the extent to which the tool measures the phenomenon it was intended to observe. This begins during the developmental phase wherein preliminary questions are usually formulated based on qualitative interviews, published literature and field observations. The questions are then presented to an expert panel who rate the relevance, readability, clarity and comprehensiveness of the questions [32]. Accordingly, qualitative interviews were conducted with residents (n = 13) and their families (n = 6) to understand the dining experience in nursing homes. Full details of the study design, interview methods, and participant demographics have been described elsewhere [29]. Briefly, older adults (14 females; 5 males; M = 78 years; SD ± 10) were recruited from within nursing homes and support groups for persons with dementia. Individual interviews with residents were used wherein they were asked to describe the food and dining in their current nursing home. Transcripts underwent descriptive content analysis by two independent researchers to determine themes relevant to food service.

In addition to resident interviews, a systematic literature review [20] was conducted to identify existing questionnaires [15,21,22] together with qualitative studies [33,34,35,36,37,38,39] that explored residents’ perspectives and experiences of the food service in nursing homes. The review demonstrated that existing resident FSSQs were more than ten years old and consequently were not developed with a person-centered lens. Critical appraisal with the COSMIN^®^ Checklist further revealed that each study exhibited flaws in content and construct validity, primarily due to inadequate sample size. Further, each of the questionnaires either did not test for external reliability or did not use the statistical methodology recommended by the COSMIN^®^ guidelines. Finally, none of the existing questionnaires were developed using theoretical frameworks of consumer satisfaction.

A preliminary 35-item version of the FSSQ was drafted and reviewed by a panel consisting of experts in various aspects of aged care, dementia care, food service and statistics. The first draft included inverse items in an effort to reduce acquiescence bias [40]; however, the panel felt that switching between contexts could be confusing and increase respondent fatigue. Negative items may also impact the internal validity of the scale, reducing the Cronbach’s alpha coefficient, which may cause significant differences in the way factors load [41]. All questions were therefore reworded as positive statements as this also increases interpretability for the end user as there is no need for a complex scoring matrix.

There is no consensus regarding the most appropriate response scale for questionnaires; however, in a population where acquiescence bias is likely, using an even numbered scale removes the neutral option and requires participants to state a preference [42]. As such, the 35-items required a response on a four-point scale, i.e., none of the time, some of the time, most of the time, all of the time. Three items utilized a seven-point Chernoff faces scale instead of the 4-point scale; they were related to resident wellbeing, global satisfaction (food) and global satisfaction (food service). The FSSQ underwent two rounds of pre-testing with residents (n = 6 and n = 10) from nursing homes (n = 4) to obtain feedback on content and clarity resulting in changes to the wording on some items.

Data Collection: Nursing homes in South Australia were invited to participate in the study via email, phone or personal contact. Homes from a range of suburbs from low to high Socio-Economic Indices for Areas (SEIFA) [43] were approached to ensure broad and even representation. Residents were eligible to participate if they permanently resided in the nursing home, had been living there a minimum of four weeks and were cognitively able to complete the questionnaire. Cognition was assessed using three items (age, date of birth and year of birth), which has been shown to be appropriate where the intent is to identify an individuals’ capacity rather than diagnose the severity of cognitive impairment [44,45]. Residents who were unable to answer these questions were still invited to complete the questionnaire and their data were included if the interviewer deemed they were alert and oriented.

The occupancy lists were screened by clinical staff to eliminate individuals who had not been living in the home for at least four weeks. Eligible residents were approached, informed about the purpose of the study and provided verbal consent to participate. During the first interview residents were also invited to complete a second questionnaire (the FoodEx-LTC) [15] to allow convergence validity to be tested. Although there is no gold standard, the FoodEx-LTC was chosen as the most suitable comparator as it was the only FSSQ currently available that met the COSMIN^®^ guidelines for establishing content/face validity [20]. Questionnaires were administered by one interviewer to test intra-rater reliability and each questionnaire was checked for completion before concluding the interview, thereby reducing missing data. Participants were approached again after four weeks to complete the questionnaire a second time for the purpose of test-retest analysis. All nursing homes had a four-week menu cycle; therefore, this time-point was chosen to minimize variability in that residents were asked to repeat the questionnaire on the same week of the menu cycle on both occasions.

In addition to the demographic and wellbeing questions, the preliminary version of the questionnaire contained 35 items related to meals and dining together with a section for indicating overall satisfaction in two domains (the meals and food service). Participants were asked to respond with one of the following options: (1) none of the time, (2) some of the time, (3) most of the time, (4) all of the time. Although not provided as an option on the scale, participants also spontaneously responded with “don’t know” and “not applicable” which was noted by hand. An open-ended question was also included, “Is there anything else about the meals here you would like to talk about?”, to provide residents with an opportunity to raise items of importance not previously identified [46].

Data analysis: Statistical analysis was conducted using the Statistical Package for the Social Sciences (IBM Corp. Released 2017. IBM SPSS Statistics for Windows, Version 25.0. Armonk, NY: IBM Corp.). Sample size was determined based on the subject to item ratio of 10:1 necessary to adequately power factor analysis; in this case, 35-items required a minimum of 350 participants [40]. For other statistical tests such as convergence validity and temporal stability, the COSMIN^®^ benchmark for excellence stipulates a sample size of ≥100 for adequate power. This determined the number of residents asked to complete the FoodEx-LTC on the first visit (convergence validity) and the test questionnaire on the second visit at four weeks (temporal stability).

Descriptive statistics of participants (age, gender, length of stay) were recorded and reported as either mean (SD) or median (IQR). As the questionnaire was interviewer administered and checked for completion prior to concluding the interview, all missing data could be considered hierarchical [47]. For example, if a resident indicated they chose to eat in their room rather than the community dining room, that made subsequent questions such as “Do you have a choice in who you sit with at meal times?” redundant and marked “not applicable”. Consequently, in order to maintain the sample size and statistical power, missing values were replaced with the mean. This has been deemed an acceptable method of addressing missingness in quality of life scales where less than 50% of responses are missing [48].

Construct validity is the extent to which the tool measures the various factors or constructs associated with the phenomenon, in this case food service satisfaction [32]. This was tested using Principal Components Analysis (PCA) with varimax rotation to group highly correlated items together to form factors. The correlation matrix was visually examined to identify items displaying a weak correlation (R < 0.35), individual Measures of Sampling Adequacy greater than 0.7, a Kaiser–Meyer–Olkin (KMO) value above 0.6 and a significant Bartlett’s Test of Sphericity [49]. The number of factors to retain were determined by comparing Eigenvalues, Cattell’s Scree Plot [50] and Velicer’s Minimum Average Partial (MAP) [51].

Convergence validity can be used to examine whether a new instrument measures the same construct as an existing measure [32]. Pearson’s correlation (for normally distributed data) or Spearman’s correlation (for non-parametric data) can be used to compare the scores of the FSSQ and the FoodEx-LTC. Correlation coefficients of *r* > 0.8 are considered very strong, *r* = 0.6–0.79 are considered strong, *r* = 0.40–0.59 are considered moderate and *r* < 0.4 is considered weak [52]. Streiner, Norman and Cairney [53] suggest that for health measurement scales, correlations within the midrange of *r* = 0.4–0.8 indicate that both instruments are measuring the same construct.

Once construct validity and item reduction has occurred, tests of reliability can be conducted on the factored scale. Internal reliability assesses consistency across items within the instrument, both across the entire scale and within individual factors. In a well-constructed tool, participants should respond consistently to related items indicating high internal reliability. This can be tested using Cronbach’s alpha with coefficients of α ≥ 0.5 considered reliable in development and coefficients of α ≥ 0.7 considered excellent, as this is the recommendation for an established questionnaire [32].

External reliability assesses consistency from one user to another; one such measure is temporal stability, also called test-retest reliability, which is a measure of consistency over time. When an instrument is stable the same participants when tested under the same conditions at different time points should yield similar results [54]. A similar measure is intra-rater reliability which measures the users’ consistency on scoring or observing the same subject across multiple time points [54]. The COSMIN^®^ guideline states that Pearson’s correlation coefficient is not adequate as it does not consider systematic error. Instead, when analyzing ordinal scales, both temporal stability and intra-rater reliability should be analyzed using weighted kappa [23]. These techniques were adopted for the purpose of this study.

## 3. Results

Twenty-three individual aged care homes were invited to participate in the study; this represented 16 aged care providers: five were large organizations who operated multiple homes, the remainder were small organizations who operated individual homes. Five aged care providers agreed to participate in the study, providing access to twenty nursing homes. The number of residents living in each home ranged from 16 to 225 (median = 90, IQR = 46). The SEIFA 2011 [43] indicated nursing homes were located across the bands of advantage and disadvantage, with 50% ranked in the top five suburbs for Adelaide and 50% ranked in the lower five suburbs.

A total of 466 residents were invited to participate in the project; 66 residents (14%) declined to be interviewed, giving a response rate of 86%. Consequently, interviews were commenced with 400 residents. Eleven interviews (2.75%) were ceased by the interviewer because the resident displayed obvious signs of cognitive impairment or confusion. Two interviews (0.5%) were ceased at the residents’ request, resulting in a total of 387 surveys included in analysis. Twenty-nine residents (7.5%) did not complete the three cognitive screening items; however, they were oriented and able to answer the food service satisfaction questions clearly; therefore, their data were retained.

Descriptive statistics of the sample are included in Table 1; briefly, 115 males (29.7%) and 272 females (70.3%) participated. Most residents (n = 373) were able to provide details regarding their age which ranged between 49 and 105 years old (median = 87 years; IQR = 13). Most residents (n = 359) were able to indicate the number of months they had been living in the aged care home, which ranged between 1 and 168 months (median = 18 months; IQR = 30). Questionnaires took between 15 and 50 min to complete (mean = 18.3 min; SD 5.587).

### 3.1. Validity: Principal Components Analysis

The KMO measure for the 35-item scale was 0.873 with a significant (*p* < 0.001) Bartlett’s Test of Sphericity, indicating the scale was suitable for factoring. Using the recommendations from the correlation matrix and individual MSA scores, four items were removed from the subsequent analysis. Examination of the second anti-image correlation showed that all remaining items were above the 0.6 threshold and were therefore retained for further analysis. Using the Eigenvalues > 1.0 guide, seven factors were identified, accounting for 52.62% of the variance; however, examination of Cattell’s Scree plot and the results of Velicer’s MAP test both indicated three factors were more appropriate. The three-factor solution resulted in removal of an additional six items, culminating in a 25-item questionnaire that explains 41.53% of the variance, with a KMO measure of 0.890 and a significant (*p* < 0.001) Bartlett’s Test of Sphericity (Table 2).

### 3.2. Validity: Convergence Validity

Spearman’s correlation was conducted to assess the relationship between the FSSQ and the FoodEx-LTC. One hundred residents completed both questionnaires, and after excluding missing values the responses from 94 cases were analyzed. There was a statistically significant high correlation between the scores of both questionnaires (*r* = 0.594, 95% CI 0.430–0.718, *p* > 0.001).

### 3.3. Reliability: Internal Consistency

Cronbach’s alpha (α) for the three-factor, 25-item scale was 0.889, which is considered good. Additionally, the (α) for the individual factors also performed well: Factor One (α) = 0.893, Factor Two (α) = 0.648 and Factor Three (α) = 0.729.

### 3.4. Reliability: Temporal Stability and Intra-Rater Reliability

Weighted kappa (Kw) with quadratic weights was used to determine if there was agreement between the responses to each question across the two time points. One hundred and five residents completed the questionnaire at baseline and four weeks; with one exception all questions displayed a moderate (0.41–0.60) to almost perfect (0.81–1.00) level of agreement (*p* < 0.001) between the two time points. Question 32 (Are they willing to provide help with cutting up your food?) had such a high level of agreement that it triggered a known paradox with weighted kappa [49]; consequently, Gwet’s AC and percent agreement were also calculated. All items had a percent agreement ranging between 0.8213 and 0.9908 and exhibited a significant Gwet’s AC (*p* < 0.001) between 0.6242 and 0.9799.

## 4. Discussion

The development of this food service satisfaction questionnaire was informed by the COSMIN^®^ benchmarks for excellence [23]; as such, a combination of methods was used, which set it apart from others available to nursing homes. This is the first nursing home resident FSSQ to include all of the following aspects during design and development: (1) consumer satisfaction theories, (2) stakeholder consultation, (3) a systematic review of the literature, (4) adequate sample size to ensure statistical power and (5) robust and appropriate statistical analysis and reporting. In addition, this is the first FSSQ to consider and include aspects of person-centered care, including those relevant to external assessment and accreditation. The final version is a 25-item FSSQ that is quick to complete, acceptable on all tests of validity and reliability and, most importantly, simple for nursing home staff to use and interpret. This FSSQ can be readily translated into practice and become an effective and efficient way for nursing homes to measure resident food service satisfaction for both quality improvement and accreditation purposes.

Design and development of the FSSQ (content validity) was underpinned by consumer satisfaction theories, drawing upon fundamentals of the Expectancy-Disconfirmation Paradigm (EDP) [55] and the Importance-Performance Model (IPM) [56]. The EDP posits that individuals determine satisfaction by comparing their expectations regarding a product or service to their actual experience [55]. Accordingly, we drew upon research by Case and Gilbert who explored resident expectations regarding the food and dining experience during item generation [57]. The Importance-Performance Model (IPM) suggests that satisfaction is related to a combination of perceived importance and performance (quality) rather than expectations or values. The argument here is that when a consumer is ambivalent about a particular feature of a product or service, they may not experience feelings of satisfaction or dissatisfaction [56]. For example, residents who wish to be involved in food preparation may feel dissatisfied if not presented with this opportunity, whereas those who do not desire or value this will feel ambivalent. Together, these theories informed the creation of an easily interpreted FSSQ that captures both resident expectations and importance/performance. In addition to a solid theoretical foundation, the development phase also included qualitative interviews with stakeholders, a review of published literature, a comparison of existing tools and an expert panel. When compared with existing resident food service satisfaction questionnaires [15,21,22], no other authors combined all these elements to establish content validity.

Construct validity was established using Principal Components Analysis, implementing a strict statistical methodology to determine how many factors to retain and which items to remove, ensuring the final version was statistically robust. The result was a 25-item, three factor questionnaire that explores the major determinants of food service satisfaction as determined by residents. Factor One is related to good food and food service and contains items related to taste, temperature, food likes, dislikes and preferences. Factor Two is concerned with resident choice and food availability and Factor Three includes items related to resident participation and staff assistance. This is congruent with themes and elements identified within the qualitative research in this field [33,34,35,37,38,39,58]. Additionally, the three factors are also consistent with previous research into food service satisfaction in this setting. Case and Gilbert suggest four themes contribute to resident satisfaction: (1) meal quality (taste, temperature, appearance); (2) individualization (adequate choice, access to familiar or culturally appropriate meals); (3) dining setting (décor, staffing, adequate assistance); and (4) socialization (ability to choose dining companion) [57]. Each of these themes are reflected in the FSSQ with Factor One addressing meal quality, Factor Two addressing food choice/availability and Factor Three consolidating the elements of dining setting and socialization.

Reliability was established through several methods, including demonstrating good Cronbach’s alpha for internal consistency, good to excellent temporal stability and moderate to very good intra-rater reliability. No other resident food service satisfaction questionnaire has been able to establish reliability across these three realms. Additionally, this is the only FSSQ validation study to have used the COSMIN^®^ guidelines for choosing the most appropriate statistical methods to establish relative measures of reliability such as test-retest and intra/inter-rater reliability [23]. Finally, this study is the first to meet established guidelines for sample size, and therefore the authors feel confident that the statistical tests were adequately powered.

Interpretability does not form part of the COSMIN^®^ checklist; however, this important factor determines the usability of the questionnaire in practice and should be considered. Questionnaires that contain negatively worded items require a matrix to determine a score that can be readily interpreted. The 25-items of this pilot questionnaire were all positively worded; therefore, no complicated matrix is required to understand the outcomes. The responses can be allocated a numerical value (1 = none of the time; 2 = some of the time; 3 = most of the time; 4 = all of the time). This gives the questionnaire a maximum score of 100, allowing for the easy conversion of the data into a percent. Additionally, drawing upon the IPM theory of satisfaction allows a resident to answer “not applicable” without negatively impacting the overall percent of satisfaction. For example, if a resident has no interest in participating in meal preparation, “not applicable” can be noted and a percent can be calculated from the remaining 24 items on the questionnaire that were of relevance.

In addition to being the first psychometrically sound resident FSSQ, this is also the first to include multiple aspects of person-centered care. Appendix A demonstrates how items in the FSSQ address the four main domains of mealtime person-centered care identified by Reimer and Keller [19]. There are seven questions relating to providing choice and preference, six items relating to independence, autonomy, and participation, two items relating to respect and one item related to social interaction. Although the methodology ensured the final version was statistically robust, it resulted in the omission of some person-centered items that are important considerations. These were Q11 “Do you have a choice in when you can eat your meals?”, Q6 “Is the dining room a comfortable and inviting place at mealtimes?” and Q19 “Are you satisfied with the amount of food that you are served?” Once a summated rating scale has been factored, it is inadvisable to ‘re-insert’ deleted items as this may invalidate the PCA. As an alternative, the items can be included at the end of the questionnaire as global measures of satisfaction. Consequently, questions 6, 11 and 19 were adapted and reframed and included with the global satisfaction items ranked with a 10-point Chernoff Faces scale:

- On a scale of 1–10, how satisfied are you with the meals here?
- On a scale of 1–10, how satisfied are you with the time meals are served?
- On a scale of 1–10, how satisfied are you the community dining area?
- On a scale of 1–10, how satisfied are you with the amount of food you are served?

Finally, previous validation studies have not explored how well residents with mild to moderate cognitive impairment understand the instrument being tested. Among the residents who participated in this study, 2.7% were unable to complete the questionnaire because they appeared confused and disoriented. A further 7.5% of participants failed to accurately recall their age or date of birth, indicating some form of cognitive impairment or memory loss; however, these residents were still capable of providing feedback about the meals to the interviewer. Historically, research in this area excludes residents with cognitive impairment; however, these results suggests that many residents with mild to moderate impairment are still capable of providing feedback on their experience with the food service.

Despite the strengths outlined there are some limitations that should be taken into consideration. Although there is increased awareness regarding the importance of measuring consumer satisfaction, there remains little agreement on the construct itself. This creates problems for researchers who must first choose which satisfaction theory is appropriate, decide how to operationalize the chosen theory and then interpret and compare the results obtained [59]. As a construct, satisfaction is highly subjective; residents in the same nursing home may have varying experiences from very dissatisfied to highly satisfied, based purely on their own expectations, values and priorities. As a cross-sectional snapshot of satisfaction this questionnaire may only give consumers a limited voice to express satisfaction; however, at an organizational level, it can be a powerful way to observe changes in satisfaction over time.

Although every effort was made to ensure nursing homes were recruited from a range of socio-economic areas, the sample lacked cultural diversity and indigenous representation. Despite the lack of ethno-centric homes in our sample, the FSSQ was designed to be applicable regardless of ethnicity. For example, questions relating to food likes and dislikes, food preferences and familiar/recognizable foods can be answered by any nationality. This was to ensure the FSSQ is broadly applicable to all aged care homes regardless of the demographic of residents in their care. Nonetheless, future research should be conducted in regions that have a multicultural client base to demonstrate generalizability. Lockdowns arising from COVID-19 meant that it was not possible to administer the FSSQ pre- and post-intervention as originally intended; when conditions allow, this should be done to test responsivity to change. Finally, inter-rater reliability should be established to ensure that consistency of results is obtained when administered by different staff members.

This FSSQ was validated among older adults who permanently reside in a nursing home; as such, they are 100% reliant on the food and food service provided to them. Consequently, this questionnaire is not intended for use in community aged care settings such as day respite services. Although these services may rely on food provided by local nursing homes or organizations such as Meals on Wheels, participants are not completely reliant on the food provided and have more autonomy and control than nursing home residents. This identifies another gap in knowledge and future research should be conducted to understand the factors that contribute to the food service satisfaction of community dwelling older adults who utilize these services.

Implications: The culture shift in aged care away from a medical model and towards a person-centered model means that we must re-evaluate the way in which we have traditionally measured food service satisfaction in nursing homes. Older studies have looked at determinants of food service satisfaction in acute and short stay settings and translated those elements into residential aged care. Although certain factors such as taste, temperature and presentation are universal to meal satisfaction, the unique conditions of living in a nursing home require a different lens. Additionally, the existing instruments were developed and designed over a decade ago when the medical model was dominant, and residents were expected to acclimatize to institutionalized food [19]. Until now, food service satisfaction questionnaires have not focused on individual needs and preferences, nor asked residents if they wish to be involved in everyday activities such as meal preparation.

Consumer feedback has always been an important component during quality improvement; it makes sense to understand your consumers needs and cater for those to increase satisfaction. In the nursing home setting this is even more important as satisfaction with the food and food services has been linked to the nutritional status of residents [11]. Therefore, improving the quality of the meals may increase resident intake and help prevent unintentional weight loss, thereby reducing associated costs (e.g., nutritional supplements) to the nursing home [60]. Further, poor satisfaction of the food service leads to both economic and environmental impact, through waste [61]. Providing nursing homes with a user-friendly measure of resident satisfaction with the food and dining empowers administrators to gather meaningful data for the purposes of quality improvement, benchmarking and accreditation.

## 5. Conclusions

Satisfaction with the food and food service is strongly linked with resident well-being and quality of life; however, the limited number of FSSQs available to nursing homes are dated and assume the medical model of care. To our knowledge, this is the first FSSQ that has been designed with a person-centered lens and is able to capture aspects such as resident involvement with the food service. This is also the first FSSQ to be made readily available to nursing homes that meets or exceeds benchmarks for establishing validity and reliability. It is quick to administer, simple to use and can provide food service managers in nursing homes with accurate and effective measures of resident satisfaction with the meals and dining. Not only does this assist with internal quality improvement, but it also gives nursing home administrators and food service managers an auditable record that can assist during accreditation.

## Figures and Tables

**Table 1:** Descriptive statistics of residents who completed the questionnaire (n = 387).

	N (%)	Median (Min;Max)
Age (years)		87 (49;105)
Responses Missing data	373 (96.4)	
	14 (3.6)	
Age (stratified by years)		
<65 65–74 75–84 85–94 95+	6	
	47	
	88	
	179	
	53	
Gender		
Male Female	115 (29.7)	
	272 (70.3)	
Length of Stay (months)		18 (1; 168)
Responses Missing data	359 (92.8)	
	28 (7.2)	
Length of Stay (stratified by months)		
<12 12–23 24–35 36–47 48–60 60+	123	
	74	
	59	
	34	
	17	
	53	
Wellbeing (scale A–G)	387 (100)	
A (Very Happy) B C D (Neutral) E F G (Very Sad)	52 (13.4)	
	103 (26.6)	
	109 (27.9)	
	82 (20.4)	
	23 (5.9)	
	16 (4.1)	
	6 (1.6)	
Diet Type	387 (100)	
Normal Texture Modified Diabetic Gluten Free Other (e.g., allergies)	279 (72.1)	
	29 (7.5)	
	32 (8.3)	
	4 (1)	
	43 (11.1)	

**Table 2:** Results of the Principal Components Analysis using Varimax Rotation revealing a 25-item, three factor solution.

Items	Factors		
	1	2	3
Q26 Do they prepare the meals the way you like?	**0.792**	0.241	1
Q20 Do you receive foods that taste good to you?	**0.782**	0.024	0.013
Q22 Do you receive foods that look appetizing to you?	**0.782**	0.170	0.066
Q10 Are you served foods that you like?	**0.707**	−0.025	−0.004
Q17 Do the meals taste like they are freshly cooked?	**0.688**	−0.080	0.121
Q35 Do they appear to be well trained in providing a good food service?	**0.676**	0.195	0.158
Q25 Are they able to provide food to meet your preferences?	**0.661**	0.276	0.041
Q2 Are you satisfied with the temperature of meals served?	**0.585**	−0.094	0.127
Q28 Do they make an effort to serve food you like?	**0.547**	0.320	0.051
Q15 Do you receive a variety of foods every day?	**0.544**	0.384	−0.032
Q12 Do you receive familiar foods that you can recognize?	**0.528**	0.068	0.039
Q3 If you make suggestions to improve the food and food service, do you feel you will be listened to?	**0.482**	0.275	0.097
Q29 Are they friendly and polite when they serve food at meal times?	**0.416**	0.156	0.276
Q1 Do you have a choice in what to eat at meal times?	0.115	**0.641**	−0.221
Q14 Are you able to invite family or friends to eat with you at meal times?	−0.032	**0.591**	0.029
Q13 Are you offered vegetables every day?	0.113	**0.561**	0.205
Q21 If you are not satisfied with the meal(s) provided are you able to choose an alternative?	0.248	**0.522**	0.008
Q7 Are you offered fresh fruit every day?	0.086	**0.481**	0.157
Q24 Can you help yourself to food whenever you feel hungry?	0.076	**0.460**	0.230
Q32 Are they willing to provide help with cutting up your food?	0.076	**0.446**	0.095
Q34 Are you able to assist them with tidying up the dining area after meals (if you wish)?	0.012	0.171	**0.758**
Q30 Are you able to assist them with setting up the dining area before meals (if you wish)?	−0.091	0.205	**0.757**
Q5 Does your meal arrive quickly after you have been seated in the dining room?	0.246	−0.070	**0.509**
Q27 Are you able to assist them with preparing meals (if you wish)?	0.074	0.109	**0.493**
Q8 Are you able to leave the dining room soon after you have finished your meal?	0.104	0.016	**0.467**

Salient loadings are shown in bold text.

## Data Availability

The data that support the findings of this study are not publicly available to maintain participant confidentiality but are available from the corresponding author (M.P.) upon request.

## Appendix A

**Aspects of Person-Centered Mealtime**	**Questions Relevant to Each Aspect**
Providing choice and preference	Q1 Do you have a choice in what to eat at meal times? Q10 Are you served foods that you like? Q12 Do you receive familiar foods that you can recognize? Q17 Do the meals taste like they are freshly cooked? Q21 If you are not satisfied with the meal(s) provided are you able to choose an alternative? Q25 Are they able to provide food to meet your preferences? Q26 Do they prepare the meals the way you like?
Supporting independence, autonomy and participation	Q32 Are they willing to provide help with cutting up your food? Q24 Can you help yourself to food whenever you feel hungry? Q3 If you make suggestions to improve the food and food service, do you feel you will be listened to? Q27 Are you able to assist them with preparing meals (if you wish)? Q34 Are you able to assist them with tidying up the dining area after meals (if you wish)? Q30 Are you able to assist them with setting up the dining area before meals (if you wish)?
Showing respect	Q29 Are they friendly and polite when they serve food at meal times? Q28 Do they make an effort to serve food you like?
Promoting social interaction	Q14 Are you able to invite family or friends to eat with you at meal times?
